# 

**DOI:** 10.1192/bjb.2018.87

**Published:** 2019-04

**Authors:** Jan R. Oyebode

**Affiliations:** Professor of Dementia Care, Centre for Applied Dementia Studies, University of Bradford. Email: j.oyebode@bradford.ac.uk

This passionate book gives insights into ways to understand and support people living with dementia. It holds out a challenge to all of us to remember the impact – for better or worse – that our words and actions have on those with dementia. It highlights the imperative of acting in ways that value people with dementia and enable them to continue to make the most of their strengths; it does so by giving the reader constructive, tangible, feasible ideas.

In making his arguments, Sabat uses the biopsychosocial framework, speaking of the importance of taking a person-centred, holistic perspective to appreciate each different person and his or her communications and reactions. He argues that prejudice, stigma and misunderstanding undermine morale and damage the lives of people with dementia, leading to inappropriate regimes in care settings and disrupted confidence and relationships.

It could be argued that none of this is new. Arguments for holistic understanding of the impact of illness have been put forward for several decades, including in relation to dementia, and the need for person-centred care is already enshrined in UK and in global policies. However, the continuing shortcomings in care and the prevailing myths about dementia indicate that there is a continuing need to improve the respect and care we offer people with this disorder. One part of this is to educate successive generations of health and social care professionals about the nature of dementia and of person-centred care, which is where this book can assist.

The book provides a path into the territory of truly understanding the nature of the sometimes-slippery concept of person-centred care. Its success lies in its readability and it is easy to pick up. The text is written in accessible language and is broken into bite-sized chunks by the use of headings written in the form of italicised questions. The arguments are based on research evidence and case studies, illustrated by personal examples from the lives of people with dementia. The superficial softness of the text provides a clever medium for some challenging messages, which Sabat gently but insistently brings home. They are challenges that are hard to refuse.

